# Persistent SARS-CoV-2-positive over 4 months in a COVID-19 patient with CHB

**DOI:** 10.1515/med-2021-0283

**Published:** 2021-05-11

**Authors:** Wenyuan Li, Beibei Huang, Qiang Shen, Shouwei Jiang, Kun Jin, Ling Ning, Lei Liu, Lei Li

**Affiliations:** Department of Infectious Diseases, The First Affiliated Hospital of USTC, Division of Life Sciences and Medicine, University of Science and Technology of China, Hefei, Anhui, 230001, People’s Republic of China

**Keywords:** COVID-19, SARS-CoV-2, convalescence, coronavirus, case report

## Abstract

In recent months, the novel coronavirus disease 2019 (COVID-19) pandemic has become a major public health crisis with takeover more than 1 million lives worldwide. The long-lasting existence of severe acute respiratory syndrome coronavirus 2 (SARS-CoV-2) has not yet been reported. Herein, we report a case of SARS-CoV-2 infection with intermittent viral polymerase chain reaction (PCR)-positive for >4 months after clinical rehabilitation. A 35-year-old male was diagnosed with COVID-19 pneumonia with fever but without other specific symptoms. The treatment with lopinavir-ritonavir, oxygen inhalation, and other symptomatic supportive treatment facilitated recovery, and the patient was discharged. However, his viral PCR test was continually positive in oropharyngeal swabs for >4 months after that. At the end of June 2020, he was still under quarantine and observation. The contribution of current antivirus therapy might be limited. The prognosis of COVID-19 patients might be irrelevant to the virus status. Thus, further investigation to evaluate the contagiousness of convalescent patients and the mechanism underlying the persistent existence of SARS-CoV-2 after recovery is essential. A new strategy of disease control, especially extending the follow-up period for recovered COVID-19 patients, is necessary to adapt to the current situation of pandemic.

## 1 Introduction

The novel coronavirus, severe acute respiratory syndrome coronavirus 2 (SARS-CoV-2), which was first reported in Wuhan, China, is causing serious public health problems globally [1]. Millions of cases have already been reported and more than 2 million deaths occurred worldwide [2]. Thus, COVID-19 has become a great threat to humanity and a challenge to disease control for all the countries owing to its transmission capacity. There have already been a lot of researches about the mechanism of COVID-19. Generally, it has been universally acknowledged that the virus spike protein contains a receptor-binding domain (RBD), which can recognize the angiotensin-converting enzyme 2 while invading into human respiratory tract and alveoli, which is quite similar to SARS-CoV [3]. Herein, we present a COVID-19 patient with intermittent viral polymerase chain reaction (PCR) test positive for >4 months after his clinical rehabilitation. Informed consent was obtained from the patient for publication of the case.

## 2 Case report

A 35-year-old male Chinese businessman, who was once an asymptomatic HBV carrier, was presented with fever but without other specific symptoms on February 1, 2020. The highest body temperature was 38.4°C. He visited the local hospital, and computed tomography (CT) scan revealed pneumonia (data not shown). Then, he was sent to the fever outpatient department of the first affiliated hospital of USTC. Considering his epidemiology history, he was transferred to the Department of Infectious Diseases for quarantine as a COVID-19 suspected patient on the same day. Informed consent was obtained from the patient for publication of the case study. Before all the symptoms occurred, the patient had dinner with a friend on January 19, 2020, who visited Hubei province but did not show any symptoms.

The patient has HBsAg-positive history for 2 years without treatment. He denied other medical or medication, as well as smoking and alcoholism, history.

**Figure 1:**
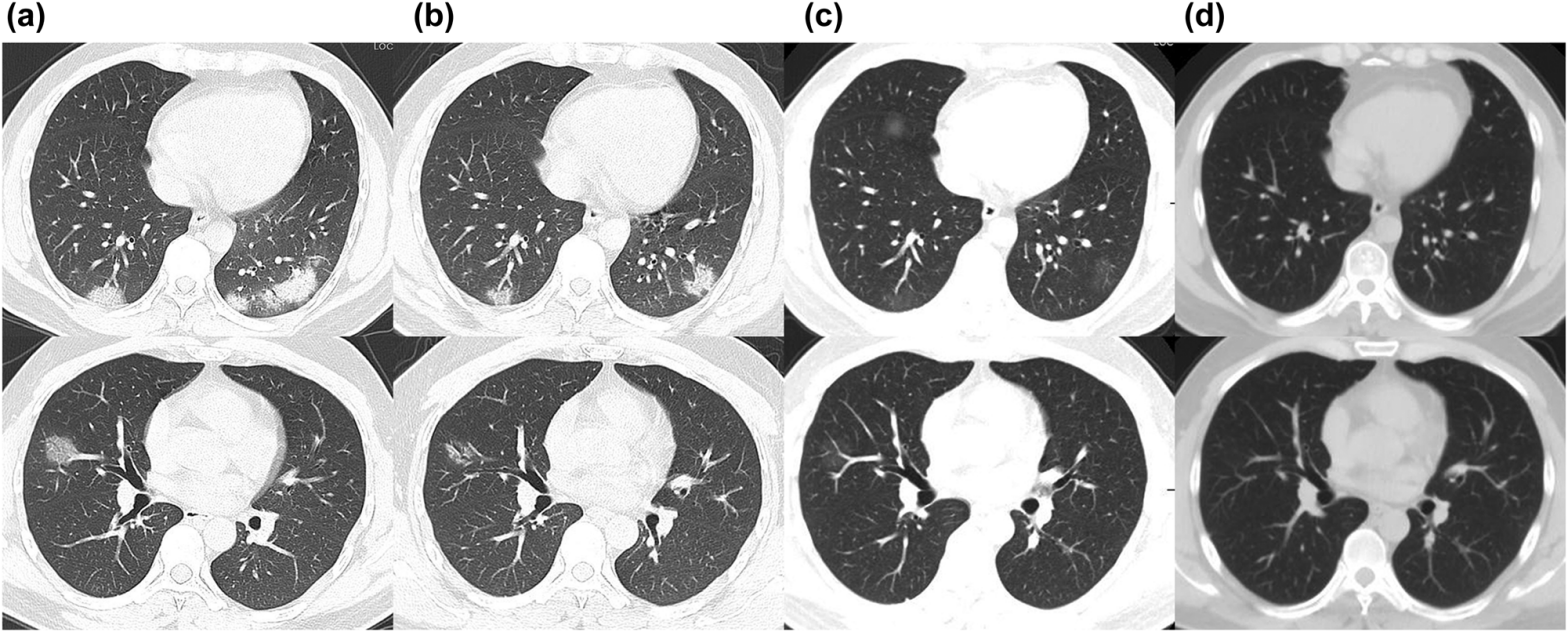
Chest CT images of the patient. (a) Multiple nodular and patchy high-density shadows on February 6; (b) Partial absorption of multiple nodular and patchy high-density shadows with ground-glass opacity on February 13; (c) Most absorbed with few parts of ground-glass opacity on March 5; (d) Normal CT image on April 29.

No specific signs were observed on physical examination. However, the oropharyngeal swabs were SARS-CoV-2 PCR-positive, as assessed by Hefei city center for disease control and prevention (CDC) on February 4, 2020 and confirmed by Anhui provincial CDC on the following day. The chest CT on February 6 showed multiple nodular and patchy high-density shadows in the bilateral lung areas (Figure 1a).

The patient was treated with lopinavir-ritonavir 500 mg po. bid, oxygen inhalation, *N*-acetylcysteine (NAC) 4.0 ivgtt. qd., and other symptomatic supportive treatment according to the COVID-19 guidelines[2,4]. The symptoms recovered satisfactorily, and the temperature dropped back to normal since day 3. Nonetheless, a chest CT scan on February 13 showed multiple nodular and patchy high-density shadows and ground-glass opacity, which was partially absorbed (Figure 1b).

After a temporary negative result on February 3, his SARS-CoV-2 PCR test was continually positive from February 4–13. Finally, he got two negative results on February 16 and 18, and hence, was discharged on February 19, according to the Chinese guidelines[5]. Interestingly, his liver function was normal during this whole period. The patient had a slight abnormal liver function on the discharge day with serum alanine transaminase (ALT) 133 IU/L. The HBV viral load was evaluated by qPCR, and diammonium glycyrrhizinate 150 mg tid was administered orally after discharge. The level of HBV-DNA on the discharge day was 3.17 × 10^6^ IU/mL, which returned back to normal later on February 21.

When the patient revisited the hepatology outpatient on March 5 after quarantine, the CT scan showed only a few ground-glass opacity, while the ALT level was elevated to 1,029 IU/L, following which he was admitted again to our department for chronic hepatitis B (CHB) (Figure 1c). Subsequently, he underwent the SARS-CoV-2 PCR screening that was reported positive on March 7. Thus, he was sent to the quarantine department, and the glutathione dose was reduced to 1.8 ivgtt. qd., while magnesium isoglycyrrhizinate 150 mg ivgtt. qd. and polyene phosphatidylcholine 15 mL ivgtt. qd. were administered along with tenofovir 300 mg qd. Daily, SARS-CoV-2 virus PCR test was performed from March 9–12 with negative results. He was then discharged on March 13 for the second time, while his ALT level was dropped back to 342 IU/L. Oral diammonium glycyrrhizinate and tenofovir were also given, and the ALT returned to the normal level on April 1.

**Figure 2:**
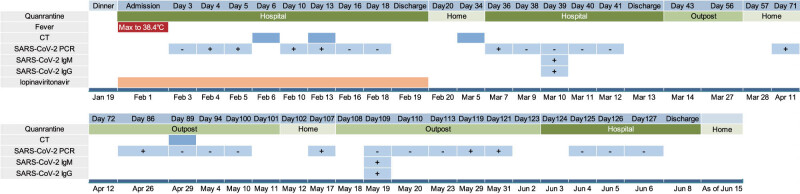
Timeline of this case after exposure.


During the months of March, April, and May, his SARS-CoV-2 virus PCR test was lingering between positive and negative, and the patient was isolated either at the quarantine outpost or at home. Chinese CDC was confused about his COVID-19 test result, and hence, he was sent to our fever outpatient on April 29 to validate the test results. Chest CT and SARS-CoV-2 PCR did not show any specific abnormality (Figure 1d). On June 3, the patient was assessed at the local hospital. With supportive treatment, the virus PCR test was negative three times on June 4, 5, and 6, respectively, following which, he was discharged on June 8. As of June 15, the patient was still under observation at home anxiously awaiting future test results (Figure 2).

**Informed consent:** Informed consent was obtained from the patient for this manuscript to be published.

## 3 Discussion

This patient presented atypical symptoms with an indirect epidemiological history of Hubei Province and was confirmed by the SARS-CoV-2 PCR-positive test. However, his SARS-CoV-2 persisted for >120 days; the symptoms and chest CT manifestation recovered adequately. As the clinical recovery of the patient is not synchronous with the existence of SARS-CoV-2 in the oropharynx, the contribution of the current antivirus therapy may be overestimated, especially for mild and moderate COVID-19 patients.

Notably, this patient was once an HBV asymptomatic carrier before COVID-19, and his transaminase level was raised markedly soon after recovery. This phenomenon indicated that SARS-CoV-2 infection might activate the virus and break the immune tolerance state, progressing the patient from HBV carrier to CHB. We also found that after Tenofovir treatment, the liver function and HBV DNA level returned to normal, but the effects on SARS-CoV-2 were not as significant as those of HBV, as deduced from the positive PCR.

As the epidemic has now been endured for over a year, many countries have faced COVID-19. Several studies have reported that recovered patients turned positive again and some of them even endured the infection for >14 days [6,7,8,9,10,11]. Some studies reported a long endurance; in one case, it was >90 days [12,13,14,15]. Although the mechanism is not yet understood, some studies indicated that immune evasion might play a significant role in the disease [9]. In addition, the RBD of SARS-CoV-2 remained dormant compared to SARS-CoV, which was beneficial for immune evasion [3]. Some other studies found that SARS-CoV-2 RNA fragments may integrate into the human genome, which may explain the situation of persistent positive of RT-PCT results in some patients [16]. Fortunately, as the SARS-CoV-2 RNA integrated into the human genome were not fully functional viral sequences, it cannot reproduce virus from human body in this way [16,17]. In addition, many studies reported that no subsequent infections were detected by these long-lasting virus-positive patients [12,15]. However, the pandemic has lasted for a year, and yet, there is no report of a case wherein PCR results are positive during the whole duration of the disease, thereby supporting the gene integration theory. Nonetheless, the underlying mechanism needs further investigation.

In the current case, the patient’s virus test was continually positive for >4 months, which is much longer than most of the cases reported previously. Thus, this could be the first case of COVID-19 lasting over 120 days. Although the virus PCR test was persistently positive, in this case, his father, who took care of him during the 14-day home quarantine, was not infected and tested negative in the PCR test. Thus, the virological status of the patient seems to be irrelevant to both the prognosis and the contagiousness. Nonetheless, the contagiousness of the recovered patients needs further investigation.

Since the viral load in the asymptomatic and symptomatic patients is similar [18], an early discharge might be problematic for both disease tracing and patient quarantine [19]. Thus, the local quarantine outpost needs three times negative results for virus tests conducted independently for discharge. If the long-lasting virus status in recovered patients is not just a rare case, disease control is challenging. A prolonged shedding time after negative coronavirus tests and multiple reexamination should also be considered. Currently, the SARS-CoV-2 virus test has been included in the routine return visit test with chest CT, blood test, and other relevant tests. The screening of coronavirus should be enforced as a regular test of admission in the hospital, as is the norm in most of the Chinese hospitals. Virus screening in the whole community or even the whole city may be carried out for asymptomatic patients, if necessary.

As the duration of the virus staying in the body is longer than expected, and a small proportion of the SARS-CoV-2 virus carrier population may become the norm, further investigation of the contagiousness of SARS-CoV-2 and new strategy of disease control are essential.

In conclusion, the contribution of antivirus therapy may be limited to mild and moderate COVID-19 patients. The prognosis of COVID-19 patients might be irrelevant to the virus status. The evaluated contagiousness of recovered patients and understanding the persistent existence of SARS-CoV-2 after recovery need to be investigated. Thus, a novel approach of disease control is necessary; extending the follow-up period for recovered COVID-19 patients could be feasible in the wake of the pandemic.

## Abbreviations

COVID-19: coronavirus disease 2019
HBV: hepatitis B virus
PCR: polymerase chain reaction
SARS-CoV-2: severe acute respiratory syndrome coronavirus 2
